# Intranasal immunization with *O*-2′-Hydroxypropyl trimethyl ammonium chloride chitosan nanoparticles loaded with Newcastle disease virus DNA vaccine enhances mucosal immune response in chickens

**DOI:** 10.1186/s12951-021-00983-5

**Published:** 2021-08-11

**Authors:** Kai Zhao, Beini Sun, Ci Shi, Yanwei Sun, Zheng Jin, Gaowei Hu

**Affiliations:** 1 Institute of Nanobiomaterials and Immunology, School of Life Science, Taizhou University, Taizhou, 318000 China; 2 Key Laboratory of Microbiology, College of Heilongjiang Province, School of Life Science, Heilongjiang University, Harbin, 150080 China; 3 Key Laboratory of Chemical Engineering Process and Technology for High-Efficiency Conversion, College of Chemistry and Material Sciences, Heilongjiang University, Harbin, 150080 China

**Keywords:** Newcastle disease virus, DNA vaccine, *O*-2ʹ-Hydroxypropyl trimethyl ammonium chloride chitosan microparticles, Intranasal delivery, Mucosal immunity

## Abstract

**Background:**

There has been a great interest in developing strategies for enhancing antigen delivery to the mucosal immune system as well as identifying mucosal active immunostimulating agents. To elevate the potential of *O*-2ʹ-Hydroxypropyl trimethyl ammonium chloride chitosan (O-2ʹ-HACC) as an adjuvant and mucosal immune delivery carrier for DNA vaccine, we prepared the O-2ʹ-HACC loaded with Newcastle disease virus (NDV) F gene plasmid DNA and C3d6 molecular adjuvant (O-2ʹ-HACC/pFDNA microparticles).

**Results:**

The O-2ʹ-HACC/pFDNA exhibited a regular spherical morphology with a particle size of 202.3 ± 0.52 nm, a zeta potential of 50.8 ± 8.21 mV, encapsulation efficiency of 90.74 ± 1.10%, and a loading capacity of 49.84 ± 1.20%. The plasmid DNA could be sustainably released from the O-2ʹ-HACC/pFDNA after an initial burst release. Intranasal vaccination of chickens immunized with O-2ʹ-HACC/pFDNA not only induced higher anti-NDV IgG and sIgA antibody titers but also significantly promoted lymphocyte proliferation and produced higher levels of IL-2, IL-4, IFN-γ, CD4+, and CD8 + T lymphocytes compared with the NDV commercial live attenuated vaccine. Intranasal delivery of the O-2ʹ-HACC/pFDNA enhanced humoral, cellular, and mucosal immune responses and protected chickens from the infection of highly virulent NDV compared with the intramuscular delivery.

**Conclusions:**

Collectively, our findings indicated that the O-2ʹ-HACC could be used as a vaccine adjuvant and delivery system for mucosal immunity and have an immense application promise.

**Graphic Abstract:**

**Figure:**
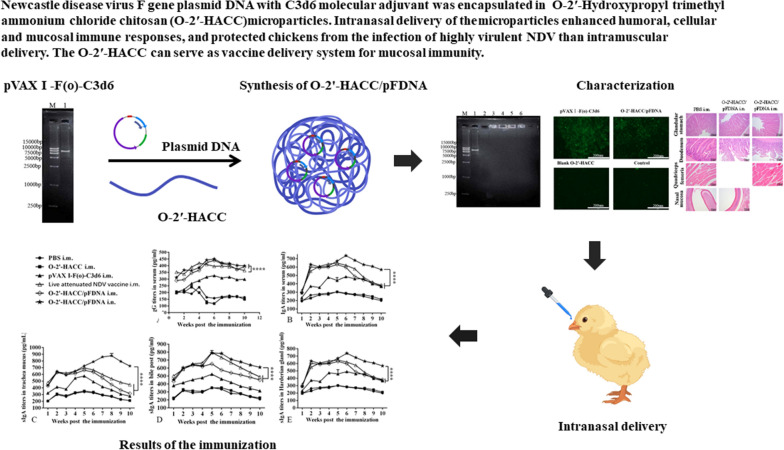

## Background

As an important part of the body's entire immune network, the mucosal immune system plays an active and important role in fighting infections [1]. The mucosal immune response can be improved by selecting the optimal immunization route, vaccine adjuvant, and delivery system [2]. Mucosal vaccination not only induces a corresponding immune response at the site of inoculation but also produces a corresponding immune response in other distant mucosal tissues. The nasal mucosa is the first part to contact the inhaled antigen, nasal mucosal immunity can induce a stronger mucosal immune response and higher systemic immune responses in the distant mucosal tissues [3, 4], and thus intranasal vaccination is considered a more favorable mucosal immune route.

Newcastle disease (ND) is an acute and highly contagious disease caused by the Newcastle disease virus (NDV) [4, 5]. Vaccination is currently the most economical and effective way to prevent ND [6]. Compared with the traditional vaccine, the DNA vaccine has great advantages and potentials, such as higher safety, better genetic stability and immune effect, simple production, convenient storage, and transportation. However, the administration of DNA vaccine is given through an intramuscular injection (i.m.), and several studies have shown that DNA vaccines don’t effectively deliver antigen to antigen-presenting cells (APCs) after i.m. Therefore, this leads to a strong immune response that can’t be induced [7, 8]. Additionally, DNA vaccine has also been limited in clinical applications due to i.m., high dose, low bioavailability, and immunogenicity [9]. Various strategies have been considered to enhance the mucosal immune response by using the suitable vaccine adjuvant, specific targeting of ligands, delivery system, and so on. Suitable vaccine adjuvant and delivery system in DNA vaccines can improve the immunogenicity, induce stronger immune responses, and reduce the dosage and production cost of vaccine in populations responding poorly to vaccination [10, 11].

Viral vectors and non-viral vectors have been used as carriers to deliver genes safely and effectively. Although viral vector has many advantages for the delivery of plasmid DNA, one of the most important issues is to ensure that the plasmid DNA is not degraded by lysosomes during transport to the host cell. Moreover, the viral vector must be non-pathogenic to the human body and will not cause proliferation and spread in the environment [12]. Compared with the viral vector, the non-viral vector has some advantages, including no infectivity, low immunogenic response, safety, high gene capacity, stability, and no carrier capacity limitation, and it is easy to prepare in large quantities [13, 14]. Non-viral gene delivery system generally consists of naked DNA delivery, lipid-based delivery, and polymer-based delivery. Cationic polymer, which electrostatically interacts with plasmid DNA to neutralize its negative charge and condense the plasmid DNA into nanosized particles, generally serves as a gene delivery system. Cationic polymer nanoparticles can protect the plasmid DNA from enzymatic degradation and facilitate cellular uptake. Intramuscularly administered polyvinyl alcohol/plasmid DNA formulation results in a significant increase in the number and distribution of the reporter-gene expressing cells in rats compared with the naked plasmid DNA [15]. Biodegradable, non-antigenic polymer-based microspheres/nanoparticles have many advantages as a vaccine adjuvant and delivery system. Our previous studies have shown that cellular, humoral, and mucosal immune responses can be elicited to antigens encapsulated in, or conjugated onto polymer-based microspheres/nanoparticles [16, 17].

Since the particle size of microspheres/nanoparticles is comparable to that of the pathogen, suitable microspheres/nanoparticles can pass through the interstitial space and capillaries to reach a site that is difficult to administer, and have many advantages, such as controlling drug release, protecting the drug from degradation or leakage, and targeting administration. Therefore, microspheres/nanoparticles can significantly improve the delivery efficiency of plasmid DNA. At present, biodegradable nanomaterials for preparing polymer-based nanoparticles mainly include chitosan and its derivatives, hyaluronic acid and sodium alginate. Among them, chitosan, the main derivative of chitin, is a linear polymer consisting of repeating units of β-(1 → 4)-2-amino-2-deoxy-D-glucopyranose units and has been proved to be a safe and non-toxic delivery carrier, and chitosan and its microspheres/nanoparticles have been broadly used as drug/vaccine delivery vectors due to their safety, non-toxicity, biocompatibility, biodegradability and sustained release in industrial and technological applications [18, 19]. However, the poor solubility of chitosan greatly restricts the application scopes and fields of chitosan. One of the strategies to improve the solubility of chitosan is to modify the structure of chitosan by the addition of hydrophilic functional groups [20]. Therefore, water-soluble chitosan derivative-based nanoparticles as a vaccine adjuvant and delivery vector have become novel vaccine/drug delivery systems. In our previous study, *N*-2-Hydroxypropyl trimethyl ammonium chloride chitosan (N-2-HACC) with good water solubility has been prepared, and the N-2-HACC is used to deliver DNA vaccine, achieving good results [21]. We have synthesized *O*-2'-Hydroxypropyl trimethyl ammonium chloride chitosan (O-2′-HACC) with good water solubility [22], which overcomes the reduction of crosslinking points during the formation of N-2-HACC resulting in the use of polyelectrolyte complex method to prepare nanoparticles. In the present study, we successfully prepared the O-2ʹ-HACC loaded with Newcastle disease virus (NDV) F gene plasmid DNA with C3d6 molecular adjuvant (O-2′-HACC/pFDNA microparticles), the positively charged O-2′-HACC and negatively charged pFDNA were attracted together by the charge, and the excess O-2′-HACC was coated outside to form particles. Because of a higher proportion of positively charged components, the O-2′-HACC/pFDNA microparticles had a zeta potential of 50.8 ± 8.21 mV. Moreover, we investigated the intranasal administration of the microparticle vaccine, and the results showed that the microparticle vaccine had good mucosal adhesion and huge potential for mucosal immunity. The prepared microparticle vaccines were termed O-2'-HACC/pFDNA in our current work. Safety is especially important for a delivery carrier, and some studies have indicated that the cellular damage caused by nanoparticles should be of great concern [23–26]. In our and others' previous work, it has been proved by both in vitro and in vivo cytotoxicity tests that chitosan-based nanoparticles are safe and can be well degraded within a certain concentration range [16, 21, 27–29]. In the present study, we focused on the side effect of the O-2'-HACC/pFDNA, and the results of in vitro and in vivo cytotoxicity showed that the O-2'-HACC/pFDNA was safe within a certain concentration range.

## Results

### Characterization of the O-2'-HACC/pFDNA

**Fig. 1:**
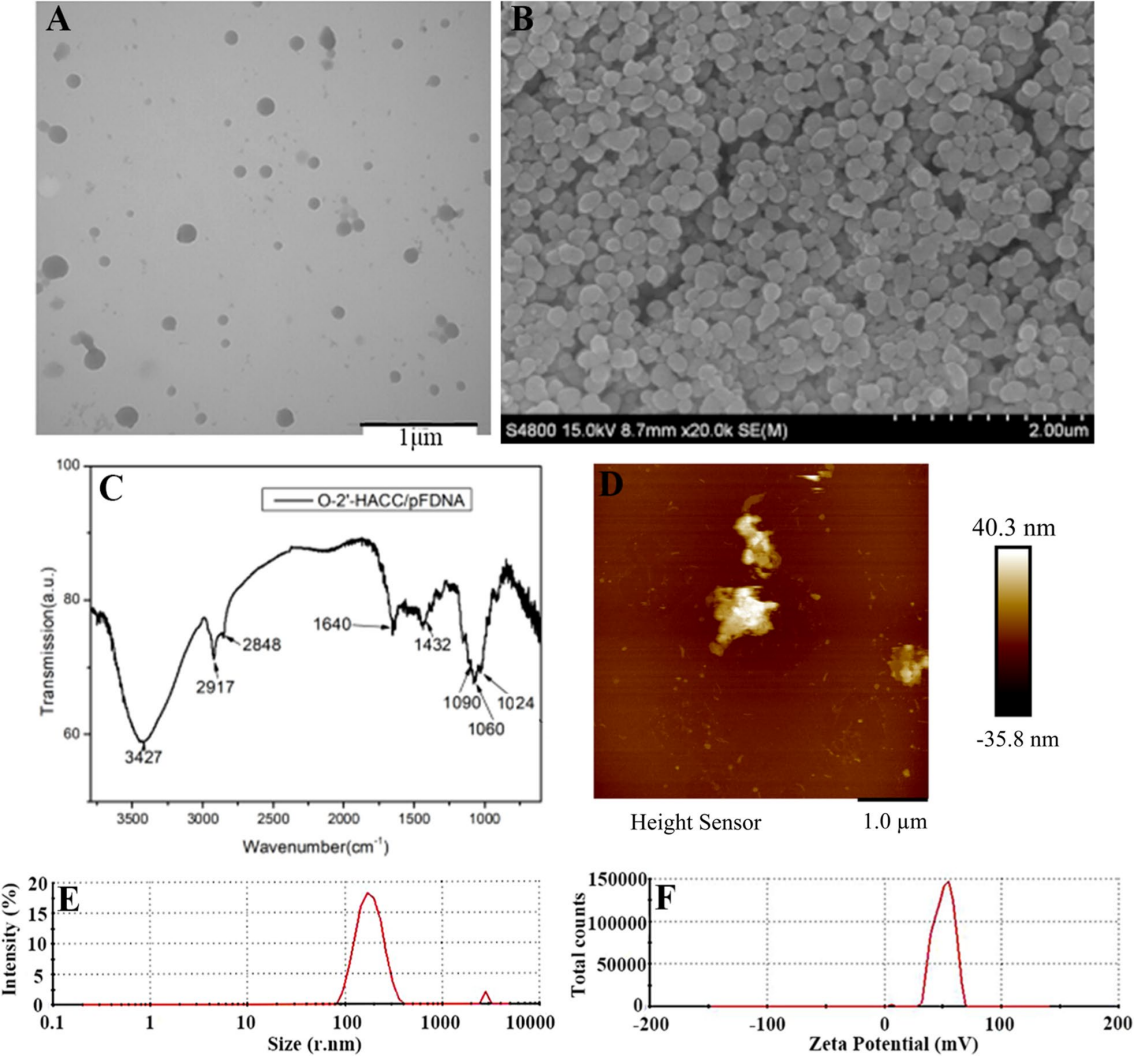
Characterization of the O-2ʹ-HACC/pFDNA. **A** TEM image of the O-2ʹ-HACC/pFDNA; **B** SEM image of the O-2ʹ-HACC/pFDNA; **C** FT-IR spectroscopy of the O-2ʹ-HACC/pFDNA; **D** AFM image of the O-2′-HACC/pFDNA; **E** Particle size of O-2′-HACC/pFDNA; **F** Zeta potential of O-2′-HACC/pFDNA

**Fig. 2:**
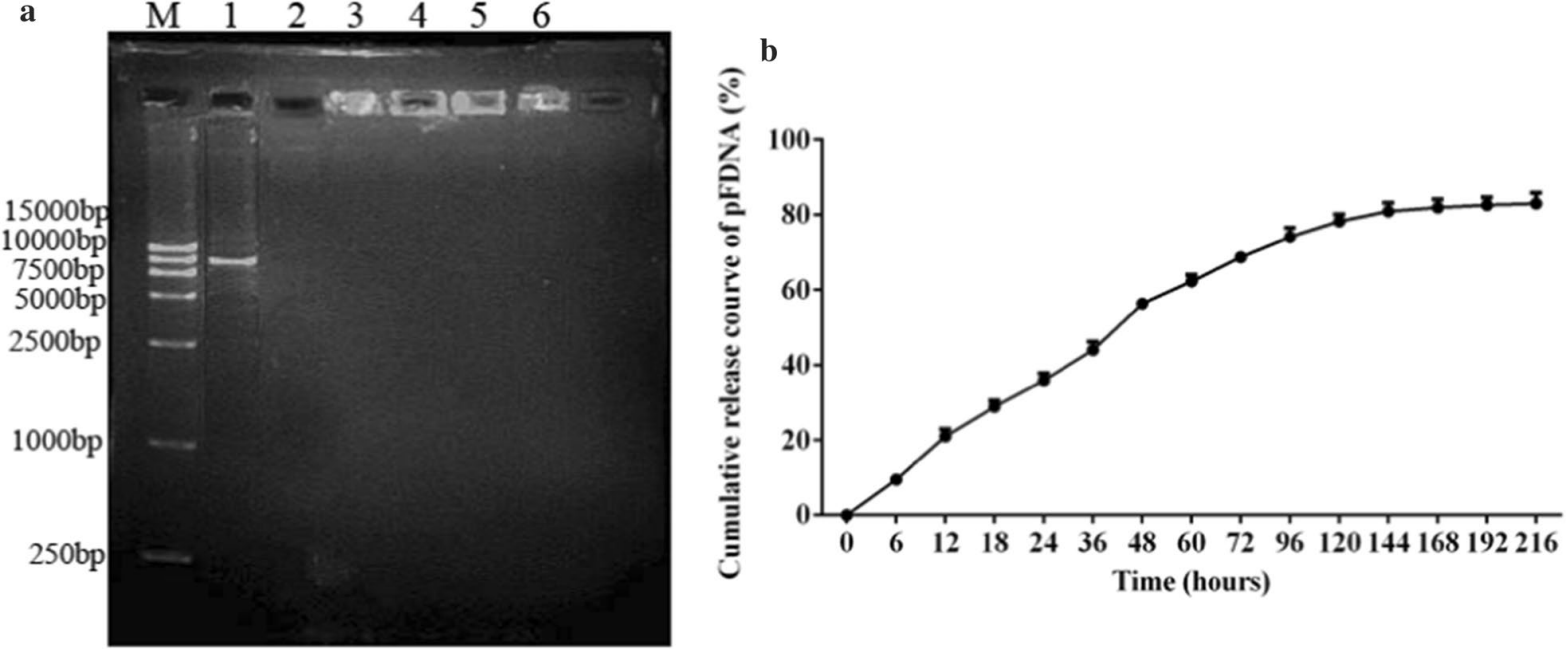
Stability and in vitro release analysis of the plasmid pVAX I-F(o)-C3d6 after encapsulation in the O-2'-HACC. **A** *DNase* I protection of the pVAX I -F(o)-C3d6, M: DL 15,000 Marker, Lane 1: pVAX I -F(o)-C3d6, Lane 2: *DNase* I acts on the naked DNA for 30 min, Lane 3–6: *DNase* I acts on the O-2′-HACC/pFDNA for 30, 60, 120 and 180 min; **B** In vitro release profiles of the O-2′-HACC/pFDNA in PBS solution (pH = 7.4). Data were presented as the mean ± SD deviation (n = 3)

O-2'-HACC/pFDNA showed a regular spherical shape, smooth surface, and good dispersion (Fig. 1A). The scanning electron microscopy (SEM) (Fig. 1B) reveals that we got a conclusion similar to the transmission electron microscopy (TEM) (Fig. 1A). The FT-IR spectroscopy of O-2 '-HACC/pFDNA (Fig. 1C) and O-2' -HACC was similar [22]. the amino peak had a slight blue shift, indicating that the electrostatic force eliminated parts of the internal hydrogen bonding. Figure 1D shows that a small amount of plasmid DNA was adsorbed around the microparticles, and the aggregation of plasmid DNA around the microparticles was conducive to the carrier particles to be the plasmid DNA wrapped into the microparticles, thus, the exogenous genes could be transported to the cell interior through cell phagocytosis. The average particle size of the microparticles was 202.3 ± 0.52 nm (Fig. 1E), the zeta potential was 50.8 ± 8.21 mV (Fig. 1F), the encapsulation efficiency (EE) was 92.27 ± 1.48%, and the loading capacity (LC) was 50.75 ± 1.35%.

### DNase I protection assay

Figure 2A shows that the integrity of plasmid DNA in O-2′-HACC/pFDNA was maintained even if the microparticles were treated with *DNase* I for 3 h (Lane 6, Fig. 2A), while the naked plasmid DNA was completely degraded by *DNase* I after 30 min (Lane 2, Fig. 2A). The results demonstrated that the plasmid DNA in O-2'-HACC/pFDNA could be protected from degradation.

### In vitro* release of O-2'-HACC/pFDNA*

Figure 2B shows that between 0 and 36 h, the release amount of plasmid DNA in O-2'-HACC/pFDNA reached 44.00 ± 1.80%, which was a process of burst release; between 36 and 120 h, the release amount reached 78.22 ± 1.60%; and after 120 h, the release of the plasmid DNA was gentle, which reached 82.97 ± 2.30%. In vitro release indicated that the O-2'-HACC could serve as a delivery vector for the sustained and slow release of DNA vaccine.

### Safety of the O-2'-HACC/pFDNA

**Fig. 3:**
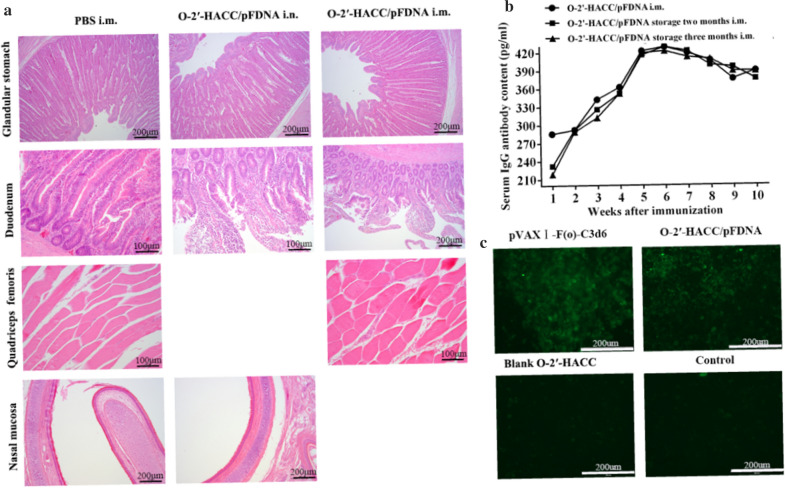
Safety analysis, in vitro fluorescence expression, and storage stability of the O-2'-HACC/pFDNA. **A** Histopathological analyses of glandular stomach, duodenum, quadriceps femoris and nasal mucosa; **B** In vitro expression of the O-2'-HACC/pFDNA in 293 T cells assayed by indirect immunofluorescence (× 40); **C** After storage stability of the O-2'-HACC/pFDNA for 2 and 3 months at room temperature, IgG titers in serum post-immunization

The survival rate of chicken embryo fibroblasts in O-2'-HACC/pFDNA was 90.48 ± 2.14%, and no significant change in cell morphology was observed compared with the control cells (*P* > 0.05). In vivo cytotoxicity analysis showed that the chickens immunized with the O-2'-HACC/pFDNA by intramuscular injection (i.m.) or intranasal administration (i.n.) were normal in terms of feeding, drinking, mental state, body weight, and inoculation sites, and there was no morbidity and mortality, indicating that the O-2'-HACC/pFDNA was safe. Histopathological analysis showed that glandular stomach, duodenum, quadriceps femoris, and nasal mucosa were intact and had no lesions (Fig. 3A). These findings indicated that the O-2'-HACC had little cytotoxicity as a delivery vector, and the O-2'-HACC/pFDNA had a higher safety level.

### Stability of the O-2'-HACC/pFDNA

The O-2'-HACC/pFDNA was a milky white powder, loose and spongy. The morphology of the microparticles was not changed after stored at room temperature, 4t, and −20℃ for 3 weeks, while there was a slight shrinkage after stored at 37℃ for 3 weeks, indicating that the O-2'-HACC/pFDNA had good storage stability and could be stored at the room temperature for a long period. Figure 3B shows that after the O-2'-HACC/pFDNA was stored at room temperature for 2 and 3 months, serum IgG antibody titers in chickens of the O-2'-HACC/pFDNA i.m. group were not significantly different from the newly prepared O-2'-HACC/pFDNA i.m. (*P* > 0.05).

### In vitro* expression of the O-2'-HACC/pFDNA*

Fluorescence was detected in the O-2'-HACC/pFDNA and pVAXI-F(o)-C3d6 groups (Fig. 3C). No fluorescence was detected in the O-2'-HACC and 293 T cell groups. These results indicated that the plasmid DNA could be efficiently encapsulated by the O-2'-HACC and expressed in vitro, indicating that the O-2'-HACC could be used for the delivery of plasmid DNA.

### Intranasal immune response

#### Serum IgG antibody titers

**Fig. 4:**
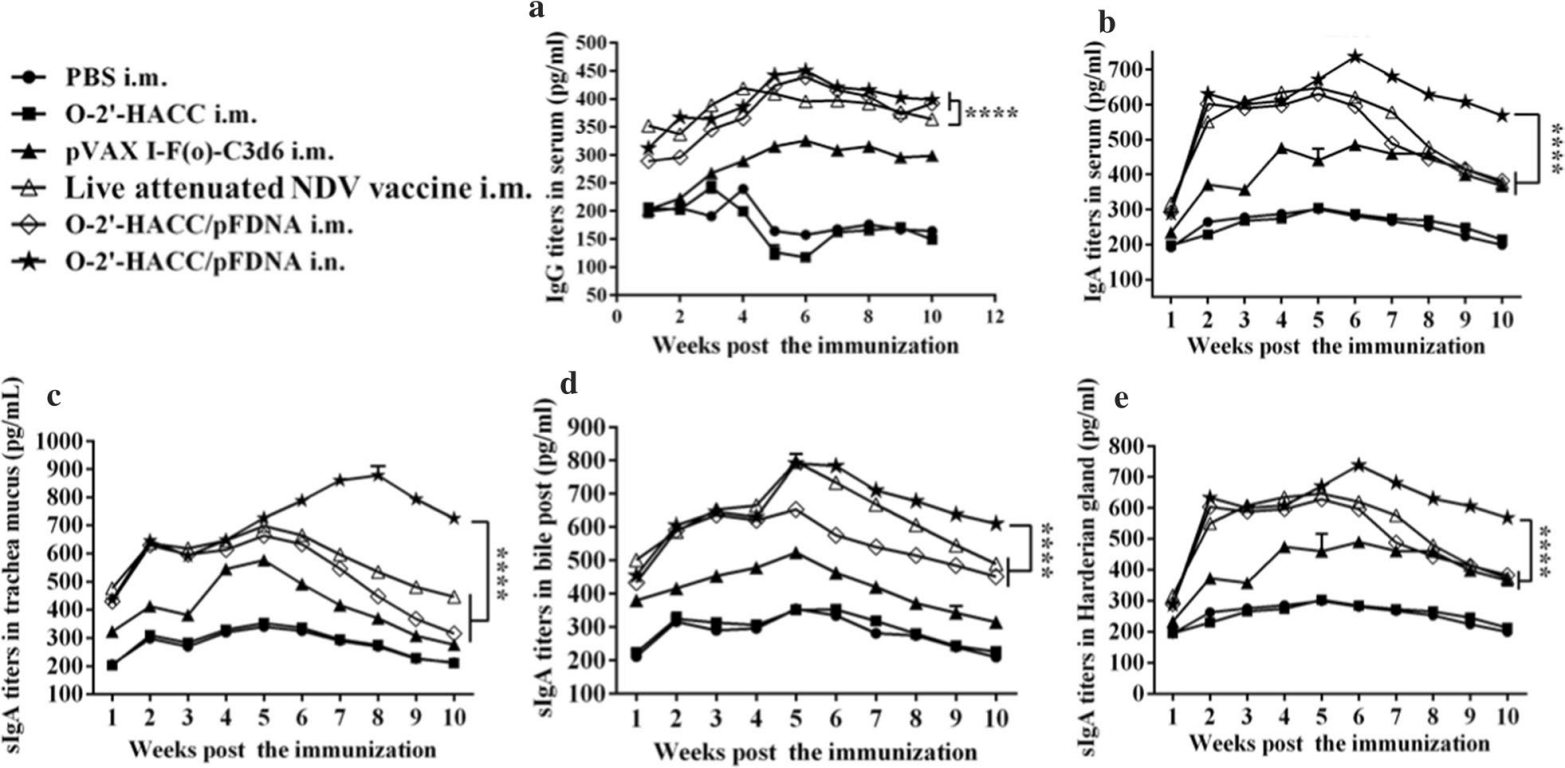
IgG and IgA antibody titers in serum (**A**, **B**), trachea mucus (**C**), bile (**D**), and Harderian gland (**E**) following administration of PBS i.m., O-2'-HACC i.m., pVAX I -F(o)-C3d6 i.m., live attenuated NDV vaccine i.n., O-2'-HACC/pFDNA i.m., O-2'-HACC/pFDNA i.n. Data were representative of three independent experiments and presented as the mean ± SD (n = 3). *P < 0.05; **P < 0.01

Figure 4A shows that at the 5th week post-immunization, the serum antibody titers were significantly increased in the pVAXI-F(o)-C3d6 i.m., O-2'-HACC/pFDNA i.m., and O-2'-HACC/pFDNA i.n. groups, and the antibody levels were higher in the O-2'-HACC/pFDNA i.m. and O-2'-HACC/pFDNA i.n. groups. Serum IgG antibody titers in the O-2'-HACC/pFDNA i.n. group peaked at the 6th week, and such higher IgG antibody levels were kept until the 10th week post-immunization. The differences between the O-2'-HACC/pFDNA i.n. and i.m. groups were not significant (*P* > 0.05), while there was a significant difference compared with the live attenuated NDV vaccine i.m. group (*P* < 0.05).

#### IgA antibody titers

IgA antibody titers in chickens immunized with the O-2'-HACC/pFDNA i.n. were significantly increased in the serum (Fig. 4B), tracheal fluid (Fig. 4C), bile (Fig. 4D), and Harderian gland (Fig. 4E) (*P* < 0.01), and the time of IgA antibody secretion was also longer than the other groups (*P* < 0.01). These results indicated that the O-2'-HACC/pFDNA i.n. induced higher IgA antibody secretion compared with the O-2'-HACC/pFDNA i.m, pVAX I -F(o)-C3d6 i.m. and live attenuated NDV vaccine i.m. (*P* < 0.01).

In addition, IgA antibody titers in the O-2'-HACC/pFDNA i.n. group were higher compared with the O-2'-HACC/pFDNA i.m, pVAXI-F(o)-C3d6 i.m., and live attenuated NDV vaccine i.m. groups (*P* < 0.01). The period of immunization protection in the O-2'-HACC/pFDNA i.n. group was longer because the O-2'-HACC increased the contact time of antigen with the mucosal surface, which effectively improved the antigen-associated lymphoid tissue and induced higher secretion levels of IgG and IgA in the body. These findings indicated that the O-2'-HACC/pFDNA produced stronger humoral immune and mucosal immune responses.

#### Lymphocyte proliferation

SI values in the PBS and O-2'-HACC groups were significantly lower compared with the pVAX I-F(o)-C3d6 i.m., O-2'-HACC/pFDNA i.m., live attenuated NDV vaccine i.m., and O-2'-HACC/pFDNA i.n. groups (*P* > 0.05). The difference between live attenuated NDV vaccine i.m. and O-2'-HACC/pFDNA i.n groups was not significant (*P* > 0.05), and after the 3rd week, the SI value in the two groups was significantly higher compared with the O-2'-HACC/pFDNA i.m. group (*P* < 0.05), showing that the O-2'-HACC/pFDNA i.n. significantly stimulated the proliferation of spleen lymphocytes (Table 1). Additionally, O-2'-HACC/pFDNA i.n. and live attenuated NDV vaccine i.m. kept strong stimulus–response to ConA until the 10th week post-immunization and produced a long-lasting immune-stimulating effect, which promoted the proliferation of lymphocytes and triggered a stronger specific immune response.

**Table 1:** Analysis of lymphocyte proliferation in SPF chickens immunized with the O-2'-HACC/pFDNA i.n., O-2'-HACC/pFDNA i.m., pVAX I -F(o)-C3d6 i.m., live attenuated NDV vaccine i.m., O-2′-HACC i.m. and PBS i.m. groups

Groups	Weeks post the immunization									
	1	2	3	4	5	6	7	8	9	10
O-2'-HACC/pFDNA i.n	0.604 ± 0.014^a^	2.490 ± 0.038^a^	3.213 ± 0.018^a^	4.141 ± 0.012^a^	4.410 ± 0.011^a^	4.384 ± 0.056^a^	4.360 ± 0.045^a^	3.930 ± 0.054^a^	3.228 ± 0.042^a^	2.983 ± 0.034^a^
O-2'-HACC/pFDNA i.m	0.568 ± 0.003^a^	2.350 ± 0.046^a^	3.086 ± 0.047^b^	3.705 ± 0.022^b^	4.145 ± 0.110^b^	3.756 ± 0.108^b^	2.668 ± 0.032^b^	2.345 ± 0.049^b^	1.986 ± 0.019^b^	1.639 ± 0.010^b^
Live attenuated NDV vaccine i.m	0.674 ± 0.008^a^	2.660 ± 0.015^a^	3.368 ± 0.040^a^	4.192 ± 0.016^a^	4.488 ± 0.015^a^	4.422 ± 0.012^a^	4.439 ± 0.078^a^	3.989 ± 0.014^a^	3.162 ± 0.055^a^	2.970 ± 0.031^a^
pVAX I -F(o)-C3d6 i.m	0.435 ± 0.018^b^	2.124 ± 0.006^b^	2.779 ± 0.017^c^	3.148 ± 0.047^c^	3.598 ± 0.013^c^	3.278 ± 0.017^c^	2.440 ± 0.005^c^	1.897 ± 0.009^c^	1.810 ± 0.010^c^	1.683 ± 0.011^b^
O-2′-HACC i.m	0.402 ± 0.004^b^	1.058 ± 0.060^c^	1.968 ± 0.019^d^	2.156 ± 0.011^d^	2.256 ± 0.038^d^	2.171 ± 0.012^d^	2.144 ± 0.010^d^	2.023 ± 0.040^d^	1.972 ± 0.017^b^	1.581 ± 0.037^c^
PBS i.m	0.306 ± 0.010^c^	1.301 ± 0.006^d^	1.924 ± 0.017^d^	2.085 ± 0.018^d^	2.117 ± 0.007^e^	1.905 ± 0.009^e^	1.879 ± 0.019^e^	1.839 ± 0.016^e^	1.702 ± 0.008^d^	1.459 ± 0.022^d^

Values represent mean ± SD (n = 3). Values within the same column with the different lower case letters (a–e) in the superscript indicate statistically significant differences (*P* < 0.05)

#### Cytokine levels in the blood

**Fig. 5:**
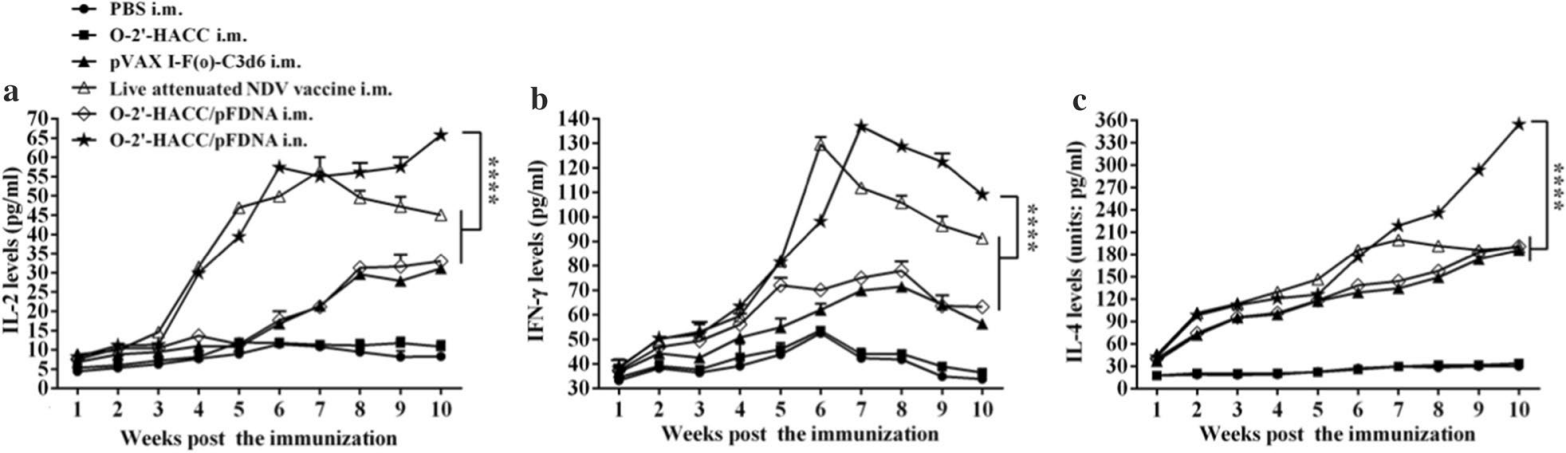
IL-2 (**A**), IL-4 (**B**), and IFN-γ (**C**) levels in the supernatant of splenocytes harvested from the SPF chickens immunized with the PBS i.m., O-2'-HACC i.m., pVAX I-F(o)-C3d6 i.m., live attenuated NDV vaccine i.n., O-2'-HACC/pFDNA i.m., and O-2'-HACC/pFDNA i.n. IFN-γ, IL-2, and IL-4 levels in the supernatant were analyzed using chicken IFN-γ, IL-2, and IL-4 ELISA kits. Results were represented as mean ± SD of three separate experiments. *P < 0.05; **P < 0.01

Figure 5 shows that the levels of IL-2, IFN-γ, and IL-4 in the blood of chickens immunized with the O-2'-HACC/pFDNA i.n. and i.m. were significantly increased compared with the pVAXI-F(o)-C3d6 i.m. and live attenuated NDV vaccine i.m. groups (*P* < 0.05), and the levels of IL-2 (Fig. 5A), IFN-γ (Fig. 5B), and IL-4 (Fig. 5C) in chickens from the O-2'-HACC/pFDNA i.n. group were higher (*P* < 0.05), indicating that the O-2'-HACC/pFDNA i.n. induced more cytokine secretion to trigger a cellular immune response.

#### *Levels of CD4* + *and CD8* + *T lymphocytes in peripheral blood*

**Fig. 6:**
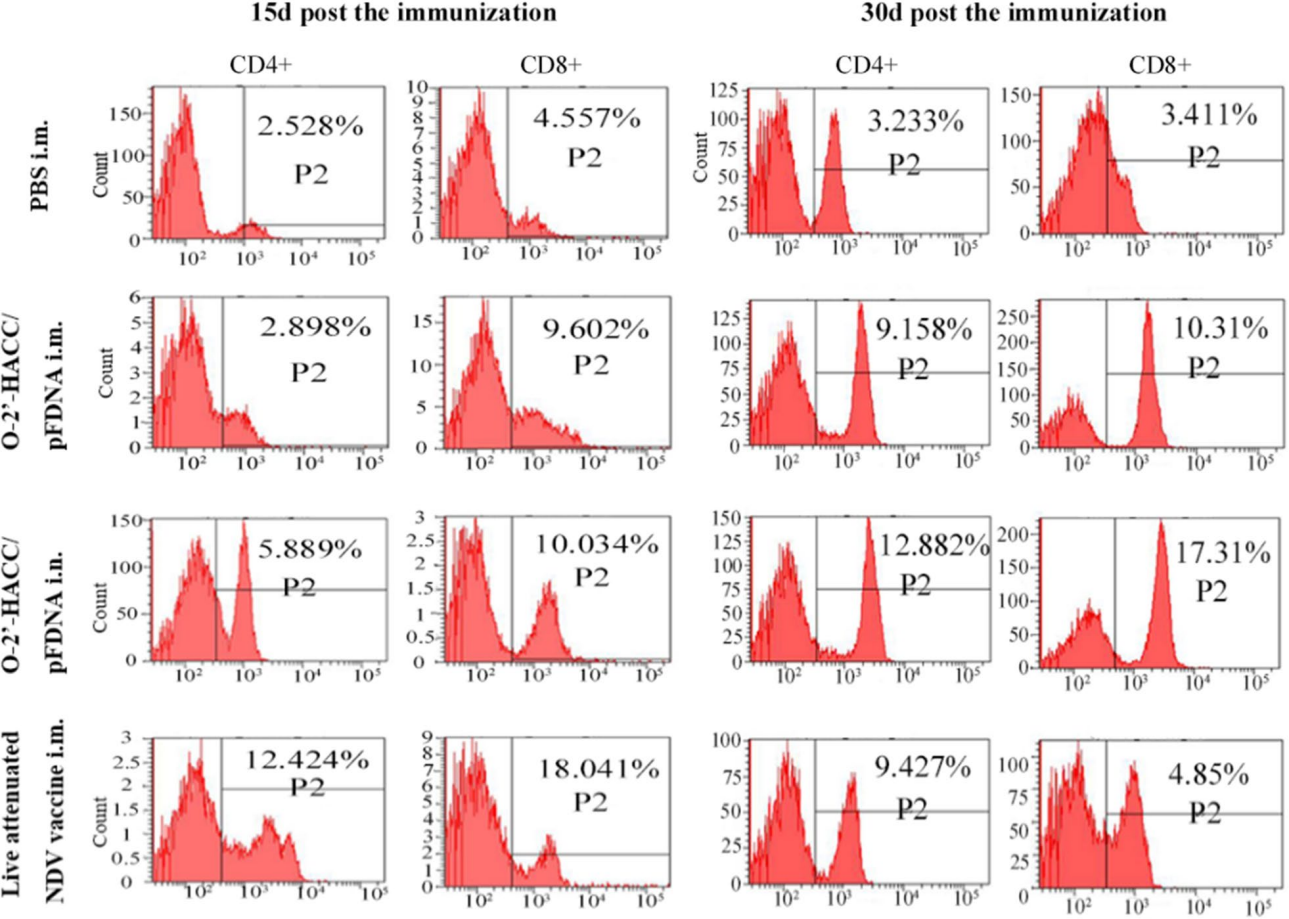
Levels of CD4 + and CD8 + T lymphocytes in peripheral blood post 15 days and 30 days post the immunization

At 15 days post-immunization, the levels of CD4 + and CD8 + T lymphocytes in the live attenuated NDV vaccine i.m. group were significantly higher compared with the PBS, O-2'-HACC/pFDNA i.n., and O-2'-HACC/pFDNA i.m. groups (*P* < 0.05) (Fig. 6). However, at 30 days post-immunization, the levels of CD4 + and CD8 + T lymphocytes in the O-2'-HACC/pFDNA i.n. group were significantly higher compared with the PBS, O-2'-HACC/pFDNA i.m., and live attenuated NDV vaccine i.m. groups (*P* < 0.05) (Fig. 6).

## Immune protective efficacy

### Serum HI antibody titers

**Fig. 7:**
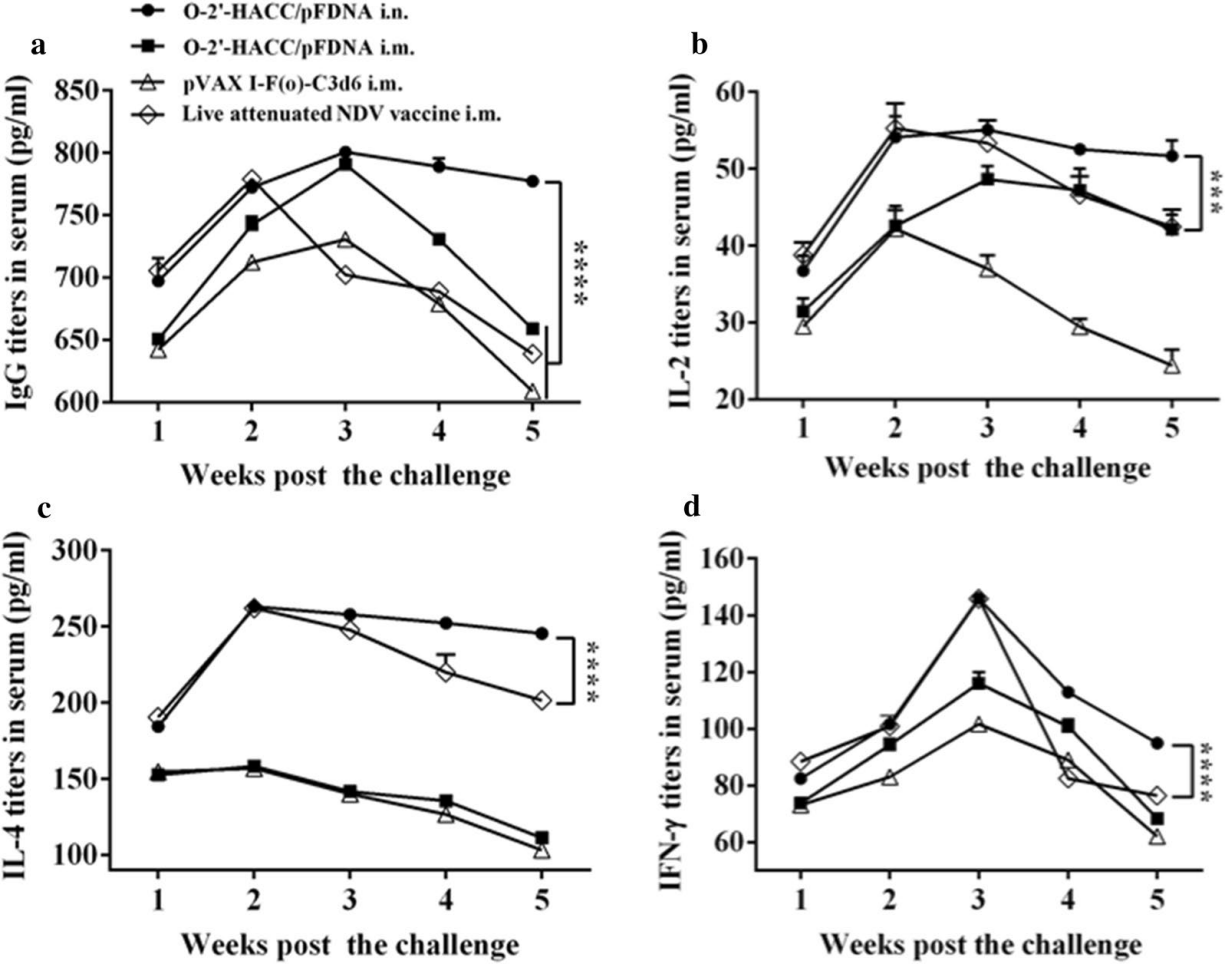
Serum IgG antibody titers (**A**) and IL-2 (**B**), IL-4 (**C**), IFN-γ (**D**) levels in the supernatant of splenocytes harvested from the immunized SPF chickens after the challenge with the highly virulent NDV strain F48E9. IFN-γ, IL-2, and IL-4 levels in the supernatant were analyzed using chicken IFN-γ, IL-2, and IL-4 ELISA kits. Results were represented as mean ± SD of three separate experiments. *P < 0.05

Anti-NDV antibody titers in chickens immunized with the O-2'-HACC/pFDNA i.n., O-2'-HACC/pFDNA i.m., and pVAX I -F(o)-C3d6 i.m. reached a peak in the 3rd week post-immunization, and the level of IgG antibody in the O-2'-HACC/pFDNA i.n. group was slightly higher compared with the O-2'-HACC/pFDNA i.m. group, while the difference between the two groups was not significant (*P* > 0.05). IgG antibody titers in the O-2'-HACC/pFDNA i.n. and i.m. groups were higher compared with the pVAX I -F(o)-C3d6 i.m. and live attenuated NDV vaccine i.m. groups (*P* < 0.05). Serum IgG antibody levels in the O-2'-HACC/pFDNA i.n. group were slowly decreased in the 3–5 weeks after the challenge and maintained a higher level (Fig. 7A).

### Changes of cytokine levels after challenge

At the 2nd week after challenge, the IL-2 content in serum in the live attenuated NDV vaccine i.m. group reached the highest value (Fig. 7B), while there was no significant difference between the live attenuated NDV vaccine i.m. and O-2'-HACC/pFDNA i.n. groups (*P* < 0.05). At the 3rd week after the challenge, the IL-2 content in the O-2'-HACC/pFDNA i.n. group reached the highest value, and it was significantly higher compared with the other groups until the 5th week (*P* < 0.05) (Fig. 7B).

At the 1–5 weeks after the challenge, the IL-4 level was significantly higher in the live attenuated NDV vaccine i.m. and O-2'-HACC/pFDNA i.n. groups compared with the pVAX I -F(o)-C3d6 i.m. and O-2'-HACC/pFDNA i.m. groups (*P* < 0.05) (Fig. 7C).

In the live attenuated NDV vaccine i.m. and O-2'-HACC/pFDNA i.n. groups, the IFN-γ content was extremely significantly higher compared with the pVAX I -F(o)-C3d6 i.m. and O-2'-HACC/pFDNA i.m. groups at the 3rd week after the challenge (*P* < 0.01), and the IFN-γ level in the O-2'-HACC/pFDNA i.n. group continued to maintain a high level until the 5th week after the challenge. From the 3rd week after the challenge, the serum IFN-γ level in the O-2'-HACC/pFDNA i.n. group was significantly higher compared with the pVAX I -F(o)-C3d6 i.m., O-2'-HACC/pFDNA i.m., and live attenuated NDV vaccine i.m. groups (*P* < 0.05) (Fig. 7D).

### Protective effect

**Fig. 8:**
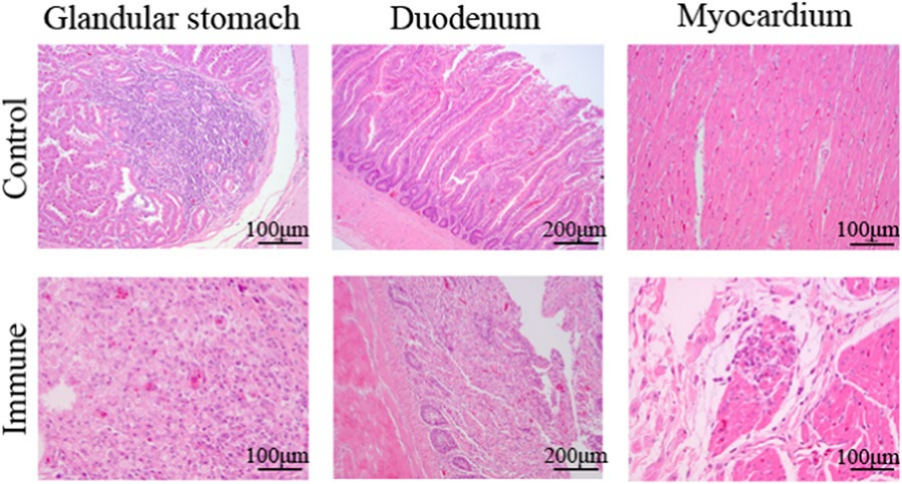
Histopathological analyses of glandular stomach, duodenum, and myocardium obtained from healthy chickens and those challenged with the highly virulent NDV strain F48E9. Tissues of the glandular stomach, duodenum, and myocardium from the PBS i.m., blank O-2’-HACC i.n., live attenuated NDV vaccine i.m., and O-2'-HACC/pFDNA i.m. and i.n. groups

Chickens in the PBS and O-2′-HACC groups died within 4–7 days after the challenge. After the challenge, two chickens immunized with the pVAX I -F(o)-C3d6 i.m. died, while chickens in the live attenuated NDV vaccine i.m., O-2'-HACC/pFDNA i.m., and O-2'-HACC/pFDNA i.n. groups didn’t die (Table 2). All the dead chickens showed the typical pathological changes of ND, such as the severe congestion of the intestinal wall and intestinal mucosa, and small bleeding spots on the surface of the glandular stomach. However, these lesions didn't appear in chickens immunized with the O-2'-HACC/pFDNA i.m., i.n. and live attenuated ND vaccine i.m. (Fig. 8).

**Table 2:** Protection efficiency of the immunized SPF chickens after challenged with the highly virulent NDV strain F48E9

Groups	Number of dead chickens/total number of chickens	Mortality (%)	Protection (%)
O-2'-HACC/pFDNA i.n	0/7	0	100
O-2'-HACC/pFDNA i.m	0/7	0	100
Live attenuated NDV vaccine i.m	0/7	0	100
pVAX I -F(o)-C3d6 i.m	2/7	28.6	71.4
O-2′-HACC i.m	7/7	100	0
PBS i.m	7/7	100	0

## Discussion

ND causes significant economic losses in the poultry industry every year. Traditional vaccines against ND have certain limitations, leading to the development of a new generation of vaccines. DNA vaccines are a new type of vaccine, which have been intensively studied in recent years. However, compared with the traditional vaccine, the DNA vaccine has some disadvantages, such as potential pathogen mutation risk and lower protection [30]. Therefore, recent vaccine studies have mainly focused on methods to improve the immune efficacy of DNA vaccines.

Biodegradable polymer-based microparticles/nanoparticles have many advantages as a vaccine adjuvant and delivery system [31]. Although plasmid DNA is quite stable in vitro, it is subject to degradation by nucleases once injected in vivo. Encapsulation of plasmid DNA in biodegradable polymer to form nanoparticles potentially offers a way to protect plasmid DNA from degradation and control plasmid DNA release [32]. Biodegradable polymers used to encapsulate plasmid DNA mainly include poly (D, L-lactic-co-glycolic) acid (PLGA), gelatin, and chitosan. Chitosan microparticles/nanoparticles have been developed for the delivery of plasmid DNA due to their cationic charge, biodegradability, biocompatibility, low toxicity, mucoadhesivity, and ability to enhance the penetration of large molecules across the mucosal surface. When DNA vaccine is encapsulated into chitosan microparticles/nanoparticles, the integrity of plasmid DNA on the mucosal surface can be protected, and the mucoadhesivity is enhanced, thereby improving its immune induction to pathogens on the mucosa [33, 34]. At present, chitosan microparticle/nanoparticle adjuvant has been applied to a variety of DNA vaccines, including human and animal infectious diseases, such as reddish body iridovirus, nodavirus, foot and mouth disease virus, and influenza virus [35, 36]. To overcome the poor water-solubility of chitosan, chitosan derivative nanoparticles used in the study, O-2ʹ-HACC, have better water solubility, biodegradability, biocompatibility, loading capacity, and mucosal adsorption compared with chitosan. Due to the presence of negatively charged regions between the cells, O-2'-HACC with positive charge can open the cell junctions in these regions and change the shape of cytoskeleton protein, allowing the O-2'-HACC to pass the mucosal epithelial cell barrier and be absorbed by M cells. Therefore, O-2'-HACC can serve as a vaccine adjuvant and delivery vector to improve the immune effect, and its nanoparticles have many advantages.

The particle size of microparticles/nanoparticles is also an important quality indicator that affects transfection and the expression efficiency of the target gene [37]. It is generally believed that microparticle vaccines between 150 and 300 nm are most suitable for transfection. If the nanoparticles are too large, it is difficult to enter the target cells [38, 39]. The particle size of O-2'-HACC/pFDNA prepared in our study was about 202.3 nm, which might help the O-2'-HACC/pFDNA to enter host cells. Moreover, the level of antibodies induced by O-2'-HACC/pFDNA was significantly higher compared with commercial vaccines, indicating that O-2'-HACC/pFDNA induced a relatively strong immune response.

Many DNA vaccines against human and animal infectious diseases have been developed [40–42]. These vaccines provide a stable and sufficient supply of antigen in transfected host cells and induce cellular immunity, mucosal immunity, and long-lasting immunity [35, 43, 44], while most DNA vaccines are injected intramuscularly or subcutaneously in clinical practice. Therefore, the mucosal immune response cannot be induced. The mucosal vaccine has many advantages over the injectable vaccine, such as simple administration, less risk of transmitting infections and ease to manufacture [45, 46]. In addition, mucosal vaccination can induce humoral and cell-mediated antigen-specific immune responses, including B cell and T cell memory responses [47].

Nasal-associated lymphoid tissue (NALT), which serves as a mucosal inductive site for immune responses against antigen stimulation in the upper respiratory tract, plays an important role in the induction of mucosal immune response, such as inducing the production of antigen-specific Th1 and Th2 cells and sIgA antibody [48–52]. Moreover, intranasal immunization can lead to the induction of antigen-specific protective immunity in both the mucosal and systemic immune compartments [50]. Therefore, intranasal immunization is expected as a vaccine against pathogens causing upper respiratory tract infections, such as NDV and influenza virus [53, 54]. In the present study, to evaluate the ability of mucosal immune response of O-2'-HACC/pFDNA, chickens were administered intranasally, and the content of sIgA antibody in tracheal fluid, bile, and Harderian gland was measured. The results demonstrated that the levels of sIgA antibody produced in the O-2'-HACC/pFDNA i.n. group were higher compared with the O-2'-HACC/pFDNA i.m. group, and the O-2'-HACC/pFDNA i.n. group exhibited a longer immune protection period, indicating that mucosal immune response was induced in mucosal inductive site for immune responses against antigen stimulation. O-2'-HACC increased the contact time of antigen with the mucosal inductive site, which effectively enhanced the uptake rate of antigen-associated lymphoid tissue. Therefore, the levels of sIgA antibody were improved, resulting in a better-induced mucosal immunity in the O-2'-HACC/pFDNA i.n. group.

T helper cells are key cells regulating humoral and cellular immunity. The functionally active region of T helper cells is divided into two cell subpopulations, Th1 and Th2 cells. Cellular immunity involves CD4 + and CD8 + T lymphocytes. CD4 + T lymphocytes can differentiate into Th1 cells or Th2 cells. Th1 cells support cellular-mediated immune responses, while Th2 cells drive humoral immune responses [55]. IL-2 mainly enhances cellular immunity, IL-4 mainly regulates humoral immunity, and IFN-γ mainly regulates immune response by participating in the differentiation of Th-type cells into Th1 type [56]. Therefore, IL-2 and IFN-γ enhance the Th1 type immune response, and IL-4 can enhance the Th2 type immune response [57]. The levels of IL-2, IL-4, and IFN-γ in the serum of chickens immunized with the O-2'-HACC/pFDNA i.n. were significantly higher, and the cytokine levels induced by the mucosal immune pathway were higher compared with the non-mucosal immune pathway, in which the O-2'-HACC/pFDNA i.n. promoted the lymphocyte proliferation and cellular response and better-induced Th1 and Th2 type responses. These findings indicated that the O-2'-HACC/pFDNA stimulated the body to produce strong cellular, humoral, and local mucosal immunity via the mucosal route.

After functional modification, chitosan derivatives can improve the various properties of chitosan, such as water solubility, stability, membrane permeability, mucosal adhesion, and controlled release. Our study provided a theoretical basis for the application of quaternized chitosan microparticles/nanoparticles as adjuvant and delivery systems for DNA vaccines in some viral infectious disease vaccines and had great potential in the field of mucosal vaccines. Despite these advantages, chitosan derivative microparticles/nanoparticles as adjuvant and delivery vectors for DNA vaccine are still in their early stages, and more clinical trials are required for verification, such as irregular distribution and low physical stability, which hinder the commercialization of chitosan. Therefore, it is highly desirable to develop a safe, efficient and targeted vaccine delivery system to prevent and control certain infectious diseases [34]. All problems will be solved shortly with the development and application of nanotechnology since one of the most attractive fields in nanotechnology is the use of nanomaterials as a vaccine adjuvant and delivery system. So many nanomaterials have been studied for the delivery of drugs, imaging, diagnostic, and vaccines. In conclusion, the use of chitosan derivative microparticles/nanoparticles has a significant impact on vaccinology with the perspective to obtain novel biological products to fight highly infectious diseases.

## Materials and methods

### Animals

A total of 210 1-day-old healthy SPF chickens were provided and raised by the Experimental Animal Center of Harbin Veterinary Research Institute, Chinese Academy of Agricultural Sciences. All animal-related procedures were approved by the Animal Ethics Committee as stipulated in the guide to the care and use of experimental animals of Harbin Veterinary Research Institute. SPF chickens were housed in the negative pressure isolator during the test. The chickens were euthanized by intravenous injection of pentobarbital.

### Preparation of the O-2ʹ-HACC/pFDNA

**Fig. 9:**
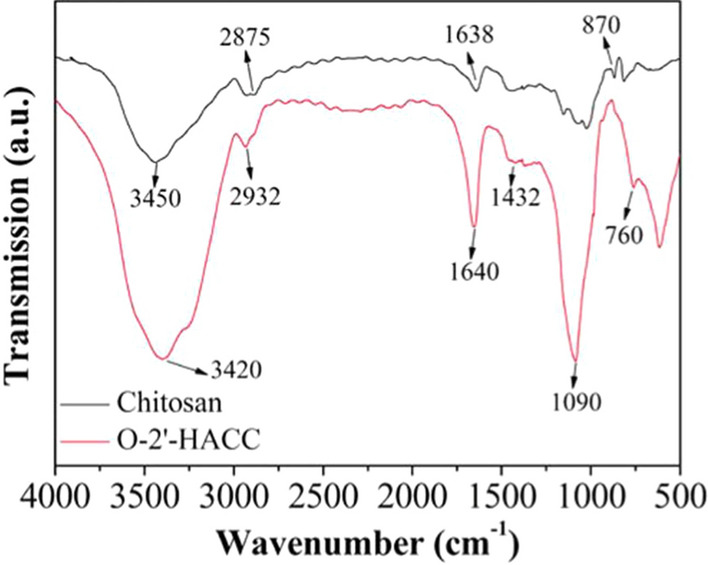
FT-IR spectroscopy of chitosan and O-2′-HACC

The O-2′-HACC loaded with NDV F gene plasmid DNA (O-2′-HACC/pFDNA microparticles) was prepared using the polyelectrolyte complex method. The water-soluble quaternized chitosan, O-2'-HACC, was synthesized as a vaccine adjuvant and delivery vector as previously described [22]. The structure of the O-2′-HACC (Fig. 9) was determined by FT-IR (Spectrum RX-1, Perkin Elmer, USA). NDV F gene eukaryotic expression plasmid pVAX-optiF with C3d6 molecular adjuvant (pVAX I -F(o)-C3d6) was constructed by our group [58].

### Characterization of the O-2'-HACC/pFDNA

To evaluate the morphological characteristics of O-2'-HACC/pFDNA, the microparticles were observed by JEM-200EX TEM (Hitachi Ltd., Tokyo, Japan), SEM (S4800, Hitachi Ltd., Tokyo, Japan), and AFM (MicroNano AFM II /III 3000, Shanghai Zhuolun Micronano Equipment Co., Ltd., China). The chemical functional groups of O-2'-HACC/pFDNA were measured by FT-IR (Spectrum RX-1, Perkin Elmer, USA). Zeta potential, particle size, and distribution of the O-2'-HACC/pFDNA were determined by a Zeta Sizer Nano ZS90 (Malvern Instruments Ltd., Southborough, MA, USA). EE and LC were determined by the formula as follows, EE (%) = (W_0_-W_1_)/W_0_ × 100%, LC (%) = (W_0_ -W_1_)/W_N_ × 100% [22], where W_0_ is the total amount of the pVAX I -F(o)-C3d6 added, W_1_ is the amount of the free pVAX I -F(o)-C3d6, and W_N_ is the weight of the O-2'-HACC/pFDNA.

### DNase I protection assay

To investigate the protection of O-2'-HACC/pFDNA against *DNase*, the O-2'-HACC/pFDNA was incubated with 10 µL of *DNase* I buffer containing 1 unit of *DNase* I (TaKaRa, Dalian, China) at 37℃ for 30, 60, 120, or 180 min. After the incubation, 5 μL of 0.5 mol/L EDTA solution was added to terminate the reaction at 65℃ for 10 min. Finally, the mixture was centrifuged at 4℃, 12,000 r/min for 20 min, and then the supernatant was taken and subjected to 0.8% agarose gel electrophoresis at 100 V for 30 min [59].

### In vitro* release of the O-2'-HACC/pFDNA*

To test the release of the pVAXI-F(o)-C3d6 from the O-2'-HACC/pFDNA, 0.1 g of the freeze-dried O-2'-HACC/pFDNA was dissolved in 2.0 mL PBS (pH 7.4), then fully mixed, and shaken at 37℃, 100 r/min for 0, 6, 12, 18, 24, 36, 48, 60, 72, 96, 120, 144, 168, 192, and 216 h. The sample was centrifuged at 4℃, 12,000 r/min for 20 min. The content of plasmid DNA in the supernatant was determined by UV spectrophotometry (ELX808, Bio-Tek, USA) at a wavelength of 260 nm. The release profile was plotted using the release time and cumulative release amount as the *X*-axis and *Y*-axis, respectively.

### Cytotoxicity and stability assay of the O-2'-HACC/pFDNA

To assess the safety of O-2'-HACC as a vaccine adjuvant and delivery system for mucosal immunity, in vitro and in vivo cytotoxicity tests were carried out. Any abnormal changes in the immunized chickens, such as feed, water drinking, mental state, body weight, clinical symptoms, morbidity, and mortality, were continuously observed for 14 days, and each dead chicken was subjected to necropsy to examine the histopathological changes by histological staining.

The animal experiment was carried out to investigate the storage stability of the freeze-dried O-2'-HACC/pFDNA stored at room temperature for 2 and 3 months. A total of 60 18-day-old healthy SPF chickens were randomly and equally divided into three groups as follows. Chickens in Group 1 were administered with the non-stored O-2'-HACC/pFDNA as a control group, chickens in Group 2 were administered with the O-2'-HACC/pFDNA stored at room temperature for 2 months, and chickens in Group 3 were administered with the O-2'-HACC/pFDNA stored at room temperature for 3 months. Each chicken received 100 μL doses via the intramuscular route. Blood samples were collected via heart from two chickens in each group at 1, 2, 3, 4, 5, 6, 7, 8, 9, and 10 weeks post-immunization, and then serum was obtained to determine the anti-NDV IgG antibody by hemagglutination inhibition (HI).

### In vitro* expression of the O-2'-HACC/pFDNA*

To verify the expression of the plasmid DNA encapsulated in the O-2'-HACC, in vitro transfection was carried out using the Lipofectamine™ 2000 reagent kit (Invitrogen, USA). Group 1 was the liposome transfection group containing 4 μg of the naked pVAX I-F(o)-C3d6, Group 2 was the O-2'-HACC/pFDNA containing 4 μg of the pVAX I-F(o)-C3d6, Group 3 was the blank O-2'-HACC as a negative control, and Group 4 was 293 T cell control group. NDV-positive serum was obtained from Harbin Veterinary Research Institute. Epifluorescence images were obtained by a fluorescence microscope (Zeiss, Germany).

### Nasal immunization

A total of 120 18-day-old healthy SPF chickens were randomly and evenly divided into six groups, and chickens in each group were separately housed in a stainless-steel isolator in a temperature- and light-controlled environment with free access to food and water. Each chicken was given an immunization dose of 100 μL containing 200 μg plasmid DNA. Chickens in Group 1 were administered with 100 μL PBS i.m., chickens in Group 2 were administered with 100 μL of O-2'-HACC i.m., chickens in Group 3 were administered with 100 μL of the plasmid DNA i.m., chickens in Group 4 were administered with 100 μL of O-2'-HACC/pFDNA containing 200 μg plasmid DNA i.m., chickens in Group 5 were administered with 100 μL of O-2'-HACC/pFDNA containing 200 μg plasmid DNA i.n., and chickens in Group 6 were administered with 100 μL of live attenuated NDV vaccine i.m. The live attenuated NDV vaccine (L/N: 200805) was provided by Harbin Pharmaceutical Group Bio-vaccine Co., Ltd.

Blood samples were collected via heart from two chickens in each group at 1, 2, 3, 4, 5, 6, 7, 8, 9, and 10 weeks post-immunization. Serum was obtained by centrifugation at 4, 3,000 r/min for 10 min, followed by measurement of the anti-NDV IgG antibody titers, the levels of IFN-γ, IL-2, and IL-4 were determined by corresponding ELISA kits (Thermo Fisher Scientific Inc., MA, USA), and the distribution of CD4 + and CD8 + T lymphocytes was tested by FACSAria flow cytometer (BD Biosciences, San Diego, CA, USA). Meanwhile, to assess the mucosal immune response, sIgA antibody titers in serum, tracheal fluid, bile, and Harderian glands were measured using the NDV IgA ELISA Kit (Rapidbio Co., Ltd., Beijing, China). Additionally, to detect the cellular-mediated immune response, splenocytes were harvested to determine the lymphocyte proliferation by MTT colorimetric assay as previously described [22].

### Protective efficacy against NDV strain F48E9

When the levels of HI antibody in serum of every immune group reached 6.0 log2 post-immunization, seven chickens were randomly selected from each group and challenged with 100 μL of viral suspension containing 10^4.5^ EID_50_/0.1 mL of F48E9 via nasal drop. Any abnormal changes, such as feed, water drinking, mental state, body weight, clinical symptoms, and mortality, were observed and recorded for 35 days. On the 7th, 14th, 21th, 28th, and 35th days after the challenge, blood samples were collected for the analysis of serum HI antibody, as well as for the contents of IFN-γ, IL-2, and IL-4. Simultaneously, the infected chickens and chickens in the negative control group were euthanized, and their glandular stomach, duodenum, and myocardium were collected to examine the histopathological changes by histological staining. Chickens were sacrificed by an overdose of the isoflurane/O_2_ mixture.

### Statistical analysis

Data were expressed as mean value ± standard deviation (SD). All experiments were repeated at least three times with at least triplicated samples in each experiment. Kruskal–Wallis one-way analysis of variance (ANOVA) was employed to evaluate the statistical differences among different groups with SPSS 19.0 software. *P* < 0.05 was considered statistically significant.

## Data Availability

All data generated or analysed during this study are included in this published article.
